# Distribution of *bla*_*CTX − M*_, *bla*_*TEM*_, *bla*_*SHV*_ and *bla*_*OXA*_ genes in Extended-spectrum-*β*-lactamase-producing Clinical isolates: A three-year multi-center study from Lahore, Pakistan

**DOI:** 10.1186/s13756-019-0536-0

**Published:** 2019-05-22

**Authors:** Samyyia Abrar, Noor Ul Ain, Huma Liaqat, Shahida Hussain, Farhan Rasheed, Saba Riaz

**Affiliations:** 1 Department of Microbiology and Molecular genetics, University of the Punjab, Lahore, Pakistan; 2 Allama Iqbal Medical College, Jinnah Hospital Lahore, Lahore, Pakistan; 3 Citilab and Research Center, Lahore, Pakistan

**Keywords:** AST, Multiplex PCR, ESBL, Phenotypic test, Molecular tests, Pakistan

## Abstract

**Background:**

Frequency of extended-spectrum-*β*-lactamase-producing clinical isolates is increasing worldwide. This is a multi-center study which was aimed to check the frequency of third-generation cephalosporin resistance and distribution of the key genetic determinants of Extended-spectrum-β-lactamase-producing Clinical isolates in Pakistan.

**Methods:**

A total of 2372 samples were processed in three tertiary care hospitals and one diagnostic research center of Lahore, Pakistan during Aug-2014 to Sep-2017. Analytical profile index (API 20-E) was used for biochemical characterization of isolates. Antibiotic susceptibility testing (AST) and third generation cephalosporin resistant (3GC-R) isolates were subjected to: double disc synergism test (DDST), combination disc test (CDST) and epsilometric test (E-test) for confirmation of ESBL-production. PCR amplification of isolates with plasmid and genomic DNA was performed. Amplicon sequences were checked for gene-variants and statistical analyses were performed to check the significance of data.

**Results:**

A total of 497/995 (50%) isolates including *Escherichia coli* 65% (*n* = 321), *Klebsiella* spp. 25% (*n* = 124) and *Pseudomonas.* 5% (*n* = 24), *Enterobacter spp.* 4% (*n* = 20) and *Acinetobacter* spp. 2% (*n* = 8) were screened as third generation cephalosporin resistant (3GC-R). Urine 56% (*n* = 278) followed by pus 20% (*n* = 99) and wound swab 6% (*n* = 29) were frequent sources. Incidence of ESBL-producers detected by combination disc test was 79% (*n* = 392). PCR revealed *bla*_*CTX − M*_ (76%) gene followed by *bla*_OXA_ (52%), *bla*_*TEM*_ (28%) and *bla*_*SHV*_ (21%) were most prevalent among ESBL-producers detected by CDST. *bla*_*CTX − M −* 1_(65%), *bla*_*OXA*_ (78%) and *bla*_*TEM*_ (57%) genes were carried on plasmids. Amplicon sequencing revealed *bla*_*CTX − M −* 15_ (75%), *bla*_*OXA −* 1_ (49%) and *bla*_*TEM −* 1*B*_ (34%) and 21 (*n* = 28) isolates carried three genes in them.

**Conclusion:**

Prevalence of ESBL-producing isolates has increased 1.13 folds during study years. Isolates had high prevalence of ESBL-encoding *bla*_*CTXM −* 15_ gene and narrow spectrum *bla*_*OXA −* 1_ and *bla*_*TEM −* 1*B*_ were also prevalent.

## Background

Multidrug resistant clinical isolates have important clinical consequence in community and hospital settings [1]. They have evolved as a global concern, exacerbated by under reporting in some regions of the world [2]. The tendency of these isolates concurrently resistant to other groups of antibiotics significantly limits the selection of antibiotics for treatment of infections [3]. The development of resistance for third generation cephalosporin is attributed to production of *β*-lactamases including extended-spectrum-*β*- lactamases (ESBLs), AmpCs and carbapenemases [4]. The most significant *β*-lactamase genes are variants of CTX-M, SHV, TEM, VEB, GES, PER, TLA and OXA which have broadened the substrate specificity against ceftazidime, cefotaxime and ceftriaxone [4, 5]. These genes have broad host range but predominantly found in *Escherichia coli* and *Klebsiella* spp. [6]. While, OXA genes are found predominantly in *Pseudomonas* spp. and *Acinetobacter* spp. [7]. Moreover, many clinical pathogens harbor more than one *β*-lactam genes [8]. Plasmid association of these genes makes them easily spreadable. Due to the diversity of these enzymes, multiplex-PCR based detection methods have become a widely used tool for epidemiological surveys [8–10].

Asian countries are highly affected by extended spectrum-*β*-lactamase-producers inducing multidrug-resistant phenotype [11–14]. Several studies have reported the community-association of ESBL-producers [11, 14, 15]. In Pakistan, an increase in the number of ESBL-associated infections has been observed in last few decades [16–21]. Lack of regular surveillance programs at national or international levels, inadequate infection control agencies, lack of facilities and inappropriate diagnostic approaches contribute to the emergence of the antibiotic resistance in bacteria [2, 10, 22]. Moreover, dissemination of these isolates in the community demands the urgent call for surveillance of resistance and molecular characterization for extended-spectrum-*β*-lactamase-producers [23]. This study was designed to check molecular epidemiology of *bla*_*CTX−M*_*, bla*_*TEM*_*, bla*_*SHV*_ *and bla*_*OXA*_ genes among ESBL-producers in Pakistani population to have a generalized view about the situation in our region.

## Materials and Methods

### Study design

This cross-sectional study was conducted at the Department of Microbiology and Molecular Genetics, University of the Punjab, Lahore in collaboration with the Department of Pathology, Allama Iqbal Medical College/ Jinnah Hospital, Lahore, Punjab Institute of Cardiology (PIC), Lahore, Doctors hospital, Lahore and Citilab and Research center, Lahore from August 2014 to September 2017. This study was approved by the ethical review board of the Citilab and Research Center, Lahore under reference: 28*th*-18 CLRC/ 28*th*.

### Bacterial Isolates

A total of 2,372 samples were processed during study period; 77 % (*n*=1835) cultures were positive and 54 % (*n*=995) gram negative non-duplicate clinical isolates from various sources were collected by standard culturing methods. Antibiotic susceptibility testing (AST) was performed according to the guidelines provided by clinical laboratory standards institute [24] by using standard antibiotic discs as mentioned in our previous study [16]. Multiple- antibiotics resistance (MAR) value was calculated as reported before [25]. *E*. coli ATCC 25922 was used as positive control and K. pneumoniae ATCC 700603 was used as negative control [26]. Analytical profile index (API 20-E) was used for biochemical characterization of isolates resistant to third generation cephalosporins.

### Phenotypic confirmation of ESBL-producers

Third generation cephalosporin resistant (3GC-R) isolates as screened by Antibiotic susceptibility test (AST) were subjected to: double disc synergism test (DDST), combination disc test (CDST) and epsilometric test (E-test) for confirmation of ESBL-production [24]. In DDST, amoxicillin (AMC 20/10μg), cefuroxime (CRO 30μg), ceftazidime (CAZ 30μg) and cefotaxime (CTX 30μg) were applied [27]**.** In CDST, CAZ (30μg) and CTX (30μg) alone and in combination with clavulanic acid (CTC (40μg) and CZC (40μg) were used [16]. All discs used were from Oxoid, Inc (Canada). For E-test, CTX / CTX+ and CAZ/CAZ+ strips from AB BIODISK MICTM were used [24].

### Molecular detection

The DNA used for multiplex-PCR was extracted by the heat lysis method [16]. In Multiplex-PCR, 2 μl whole cell lysate DNA for each isolate was used separately in 25 μl PCR-master mix and amplification primers as previously mentioned [16, 28, 29]. PCR amplification conditions were: Initial step of denaturation at 95°C for 5 min followed by 35 cycles of denaturation at 95°C for 1 min then annealing at 56°C for 1.5 min, extension at 95°C for 1 min and the final extension was done at 95°C for 10 min (Table 1).

**Table 1:** Primer sequences and amplification conditions used in this study

Target gene	Primer name	Sequence	Annealing temp (°C)	Product size (bps)	References
*bla*_CTXM-1_	CTXM1-F	GACGATGTCACTGGCTGAGC	55	500	[8]
	CTXM1-R	AGCCGCCGACGCTAATACA			
*bla*_CTXM-3_	CTXM825F	CGCTTTGCCATGTGCAGCACC	55	300	[8]
	CTXM825R	GCTCAGTACGATCGAGCC			
*bla*_SHV_	SHV-F	AGGATTGACTGCCTTTTTG	56	392	[9]
	SHV-R	ATTTGCTGATTTCGCTCG			
*bla*_TEM_	TEM-C	ATCAGCAATAAACCAGC	56	516	[9]
	TEM-H	CCCCGAAGAACGTTTTC			
*bla*_OXA_	OXA-F	ATATCTCTACTGTTGCATCTCC	56	619	[9]
	OXA-R	AAACCCTTCAAACCATCC			

### Amplicon sequencing and in-silico analysis

PCR amplified products were sequenced by Advance Bioscience International, Pakistan in collaboration with 1st Base, Malaysia [30]. Nucleotide sequence similarity searches were performed using the services of National Centre for Biotechnology Information (NCBI) (https://blast.ncbi.nlm.nih.gov/Blast.cgi). BLAST, CLUSTALX, and MEGA 7.0 software were used for sequence alignment of amplicon sequenced obtained with already submitted sequences of *bla*_*CTX−M*_*, bla*_*TEM*_, *bla*_*SHV*_ and *bla*_*OXA*_ in GenBank.

### Statistical Analysis

All statistical analyses were performed using IBM-SPSS statistics 23. Bivariate analyses were performed using chi-square test for categorical variables. All *p*-values were two sided. The percentage values included in this article are the “valid percentages,” which exclude the missing data.

## Results

### Demographic data and distribution of clinical isolates

A total of 50 % (*n*=497/995) third generation cephalosporin resistant (3GC-R) clinical isolates were found among 995 gram-negative isolates. These include 65 % (*n*=321) *Escherichia coli*, 25 % (*n*=124) *Klebsiella pneumoniae,* 5 % (*n*=24) *Pseudomonas aeruginosa* and 4 % (*n*=20) were *Enterobacter* spp. and small number of *Acinetobacter* spp. 2 % (*n*=8) were found. These isolates were obtained from urine 59 % (*n*=278), pus 20 % (*n*=99), wound swab 6 % (*n*=29), Foley’s tip 3 % (*n*=17), sputum 3 % (*n*=16), tracheal secretion 3 % (*n*=14), body fluids 2 % (*n*=10), blood 8 % (*n*=40) and HVS 1 % (*n*=4) respectively with *p*-value <0.0001. Among study population, significantly (*p*-value <0.0001) higher number of strains were isolated from males 53 % (*n*=265) compared to females 47 % (*n*=232). Age group 41-60 years was prevalent 35 % (*n*=174) followed by 21-40 years 29 % (*n*=146) (*p*-value <0.0001) (Table 2).

**Table 2:** Distribution of isolates according to different parameters from 2014 to 2017

Study year	2014	2015	2016	2017	2014–2017	Chi-score	*p-*value
Parameters							
Initial screening							
Samples Processed	400 (22)	500 (27)	500 (27)	435 (24)	1835 (48)	9.33	0.0023
Strains Screened	230 (23)	230 (29)	230 (23)	250 (25)	995 (54)	1.91	0.1671
3GC-R^a^	134 (27)	85 (23)	85 (17)	166 (33)	497 (50)	30.05	<0.0001
Third generation cephalosporin resistant isolates							
*Klebsiella* spp.	22 (18)	32 (26)	45 (36)	25 (20)	124 (25)	31.19	<0.0001
*Escherichia coli*	106 (33)	41 (13)	40 (12)	134 (42)	321 (65)	21.99	0.0012
*Enterobacter* spp.	6 (30)	12 (60)	0 (0)	2 (10)	20 (4)	15.95	0.0140
*Pseudomonas* spp.	0 (0)	24 (100)	0 (0)	0 (0)	24 (5)	74.48	<0.0001
*Acinetobacter* spp.	0 (0)	3 (38)	0 (0)	5 (63)	8 (2)	4.32	0.6335
Demographic Data							
Gender based distribution							
Male	69 (51)	47 (42)	47 (55)	102 (61)	265 (53)	11.52	0.0342
Female	65 (49)	65 (58)	38 (45)	64 (39)	232 (47)		
Age wise distribution							
< 1–20	17 (25)	27 (20)	14 (21)	10 (15)	68 (14)	32.19	0.0013
21–40	33 (23)	26 (19)	40 (27)	47 (32)	146 (29)	60.29	<0.0001
41–60	45 (26)	41 (31)	27 (16)	61 (35)	174 (35)	58.53	<0.0001
61–80	39 (37)	15 (11)	4 (4)	47 (45)	105 (21)	53.13	<0.0001
> 80	0 (0)	3 (2)	0 (0)	1 (25)	4 (1)	7.98	0.7867
Sample source							
Urine	80 (58)	150 (66)	26 (31)	16 (33)	272 (55)	18.93	0.0003
Blood	5 (4)	11 (5)	5 (6)	12 (25)	33 (7)	27.4	<0.0001
Pus	40 (29)	33 (15)	18 (21)	7 (15)	98 (20)	9.74	0.0209
Wound	2 (1)	17 (8)	11 (13)	13 (27)	43 (9)	29.26	<0.0001
Tissue	2 (1)	0 (0)	1 (1)	0 (0)	3 (1)	3.75	0.2898
Sputum	3 (2)	1 (0)	12 (14)	0 (0)	16 (3)	38.79	<0.0001
Tips	1 (1)	0 (0)	2 (2)	0 (0)	3 (1)	6	0.1116
Secretions	0 (0)	4 (2)	8 (9)	0 (0)	12 (2)	22.12	<0.0001
Fluid	3 (2)	7 (3)	0 (0)	0 (0)	10 (2)	4.02	0.2540
High vaginal swab	2 (1)	2 (1)	2 (2)	0 (0)	6 (1)	1.77	0.6215
Washings	0 (0)	1 (0)	0 (0)	0 (0)	1 (0)	1.2	0.731
Phenotypic detection tests							
Phenotypic test							
AST^b^	134 (27)	112 (23)	85 (17)	166 (33)	497 (50)	1.45	0.9975
CDST^c^	102 (26)	65 (16)	85 (21)	147 (37)	399 (80)	20.8	0.0136
DDST^d^	95 (35)	89 (33)	18 (7)	71 (26)	273 (55)	32.19	0.0002
E-test^e^	74 (24)	84 (28)	37 (12)	108 (36)	303 (61)	5.36	0.8019

Percentages are mentioned in parenthesis

^a^Third generation cephalosporin resistant

^b^Antibiotic susceptibility testing

^c^Combination disc test

^d^Double disc synergy test

^e^Epsilometric test

### Phenotypic screening and confirmation of ESBL-producers

Isolates had high resistance towards *β*-lactams including cefotaxime and cefaclor 100% (*n* = 497). While 98.6% (*n* = 490) and 96.4% (*n* = 479) isolates were resistant for cefuroxime and ceftazidime respectively. While, resistance for carbapenems was low 11% (*n* = 55). Moderate to high resistance towards aminoglycosides (67–89%) and quinolones (74–82%) was seen except amikacin 14% (*n* = 70). While isolates were quite susceptible to cefoparazone/sulbactam 6% (*n* = 30) and piperacillin/tazobactam 24% (*n* = 119). 60% (*n* = 303) of isolates had MAR-value in the range of 0.60 to 0.799, while 27% (*n* = 136) were having MAR-value of 0.8–1.0. Rest of the isolates 14% (*n* = 57) had MAR-value of 0.2–0.59. ESBL-positivity was as follows; double disc synergy test 55% (*n* = 273), combination disc test 79% (*n* = 392) and epsilometric-test showed 58% (*n* = 288). Year-wise data indicated frequency of ESBL-producers among 3GC-R has increased from 76% (*n* = 102) to 88% (*n* = 146) during study years (Table 2). *E. coli* 75% (*n* = 241), *K. pneumoniae* 80% (*n* = 99), *Pseudomonas* spp. 72% (*n* = 15) and *Enterobacter* spp. 75% (*n* = 15) had ceftazidime/ceftazidime+ MIC > 32/0.064 = 500 while 5.6% (*n* = 28) remained non-determined. Cefotaxime/cefotaxime+ > 16/0.016 = 1000 was most frequent MIC with *E. coli 64*% (*n* = 206), *K. pneumoniae 69*% (*n* = 85), *Pseudomonas spp.* 63% (*n* = 15), while 6% (*n* = 30) remained non-determined by cefotaxime/cefotaxime+ (Table 3).

**Table 3:** Minimum Inhibitory Concentration (MIC) of applied antibiotics along with clavulanic acid

Ceftazidime/ceftazidime with clavulanic acid MIC^a^ (μg/ml)						
MIC ratio	> 32/> 4 (ND)^a^	> 32/0.064 = 500	> 32/0.125 = 256	24/0.19 = 126	16/0.38 = 42.1	4/0.25 = 16
*E. coli* (*n* = 321)	13 (4%)	241 (75%)	39 (12%)	19 (6%)	3 (1%)	6 (2%)
*Klebsiella spp.* (*n* = 124)	2 (1.8%)	99 (80%)	10 (8.3%)	6 (4.6%)	7 (5.5%)	0 (0%)
*Pseudomonas spp.* (*n* = 24)	5 (21%)	17 (72%)	2 (8.3%)	0 (0%)	0 (0%)	0 (0%)
*Enterobacter spp.* (*n* = 20)	2 (10%)	15 (75%)	2 (10%)	0 (0%)	1 (5%)	0 (0%)
*Acinetobacter spp.* (*n* = 8)	6 (75%)	2 (25%)	0 (0%)	0 (0%)	0 (0%)	0 (0%)
Cefotaxime/cefotaxime with clavulanic acid MIC (μg/ml)						
MIC ratio	> 16/> 1 (ND)^b^	> 16/0.016 = 1000	12/0.023 = 521	3/0.023 = 130	8/0.125 = 64	4/0.094 = 42.5
*E. coli* (*n* = 321)	17 (5.4%)	206 (64%)	58 (18%)	17 (5.4%)	12 (3.7%)	11 (3.4%)
*Klebsiella spp.* (*n* = 124)	2 (1.8%)	85 (69%)	19 (15%)	9 (7.4%)	7 (5.5%)	2 (1.8%)
*Pseudomonas spp.* (*n* = 24)	2 (8.3%)	15 (63%)	0 (0%)	0 (0%)	2 (8.3%)	1 (4%)
*Enterobacter spp.* (*n* = 20)	1 (5%)	2 (10%)	0 (0%)	0 (0%)	0 (0%)	17 (85%)
*Acinetobacter spp.* (*n* = *8)*	8 (100%)	0 (0%)	0 (0%)	0 (0%)	0 (0%)	0 (0%)

^a^Minimum-inhibitory concentration and ^b^Not-determined

Association analysis indicated among 82% (*n* = 262) ESBL-positive *E. coli*, females were more prone to such infection with 53% (*n* = 138). While, infectivity rate was high for males with ESBL-positive *Klebsiella pneumoniae* 54% (*n* = 47) and *Enterobacter* spp. 57% (*n* = 8) (Tables 4 and 5). High frequency of ESBL-producers 36% (*n* = 140) came from age group of 41–60 years. Age associated ESBL-infectivity rate was more confined to age group 41–60 years in *E. coli* 36% (*n* = 94), *Klebsiella* spp. 38% (*n* = 35) and *Pseudomonas* spp. 33% (*n* = 8) (Table 6). Urine samples were frequent source of ESBL-phenotype among *E. coli* 65% (*n* = 171), *Klebsiella* spp. 39% (*n* = 35) and *Pseudomonas* spp. 38% (*n* = 9) (Table 5).

**Table 4:** Gender based association of infectivity among different isolates

Isolates	Gender	Number (%)	ESBL-production		Chi-score	Odds ratio	*p*-value
			Positive (%)	Negative (%)			
*E. coli* (*n* = 321)	Male	152 (47)	124 (82)	28 (18)	1.834996	1.0317 (0.5834–1.8245)	0.9146
	Female	169 (53)	138 (82)	31 (18)			
*Klebsiella spp.* (*n* = 124)	Male	68 (55)	49 (72)	19 (28)	4.08124	0.989 (0.4502–2.1746)	0.919
	Female	56 (45)	40 (71)	16 (29)			
*Pseudomonas spp.*(*n* = 24)	Male	13 (54)	10 (77)	3 (23)	0.994083	0.3333 (0.0294–3.775)	0.375
	Female	11 (46)	10 (91)	1 (9)			
*Enterobacter spp.*(*n* = 20)	Male	12 (60)	8 (67)	4 (33)	1.123626	0.6667(0.0902–4.9281)	0.6912
	Female	8 (40)	6 (75)	2 (25)			
*Acinetobacter spp.* (*n* = 8)	Male	3 (38)	2 (67)	1 (23)	0.337912	0.4000 (0.0160–10.0173)	0.5771
	Female	5 (62)	5 (100)	0 (0)

**Table 5:** Age-wise association of ESBL-production with different isolates

Isolates	Age group	Number (%)	ESBL-production		Chi-score	*p-value
			Positive (%)	Negative (%)		
*Escherichia coli* (*n* = 321)	0–20	44 (14)	41 (93)	3 (7)	0.900678	
	21–40	100 (31)	82 (82)	18 (3)	0.767442	
	41–60	120 (37)	94 (78)	26 (3)	0.045746	
	61–80	53 (17)	45 (85)	8 (6)	1.40471	
	> 80	4 (1)	0 (0)	4 (75)	14.33333	<0.0001
*Klebsiella* spp. (*n* = 124)	0–20	22 (18)	18 (82)	4 (18)	0.170543	
	21–40	38 (31)	28 (74)	10 (26)	0.450632	
	41–60	46 (37)	35 (76)	11 (24)	0.118343	
	61–80	17 (14)	10 (59)	7 (41)	3.734724	
	> 81	1 (1)	1 (100)	0 (0)	0.27907	
*Pseudomonas* spp. (*n* = 24)	0–20	6 (25)	5 (83)	1 (17)	0.093346	
	21–40	5 (21)	4 (80)	1 (20)	0.00969	
	41–60	10 (42)	8 (80)	2 (20)	0.01938	
	61–80	3 (13)	3 (100)	0 (0)	0.837209	
	> 81	0 (0)	0 (0)	0 (0)		
*Enterobacter* spp. (*n* = 20)	0–20	5 (25)	4 (80)	1 (25)	0.00969	
	21–40	5 (25)	3 (60)	2 (67)	0.968992	
	41–60	5 (25)	2 (40)	3 (150)	4.273256	0.04
	61–80	5 (25)	5 (100)	0 (0)	1.395349	
	> 81	0 (0)	0 (0)	0 (0)		
*Acinetobacter spp.* (*n* = 8)	0–20	1 (13)	0 (0)	1 (13)	3.583333	0.03
	21–40	3 (38)	1 (13)	2 (25)	3.537468	0.03
	41–60	1 (13)	1 (13)	0 (0)	0.27907	
	61–80	2 (25)	1 (13)	1 (13)	0.931202	
	> 81	1 (13)	1 (13)	0 (0)	0.27907	

*only *p*-values <0.05 are shown

**Table 6:** Association of ESBL-production with type of isolates under investigation

Isolates	Total number (%)	ESBL-production		Chi-score	*p*-value
		Positive (%)	Negative (%)		
*Escherichia coli*	321 (65)	262 (82)	59 (18)	6.792369985	.00951
*Klebsiella spp.*	124 (25)	89 (72)	35 (28)		
*Pseudomonas spp.*	24 (5)	20 (83)	4 (17)		
*Enterobacter spp.*	20 (4)	14 (70)	6 (30)		
*Acinetobacter spp.*	8 (2)	7 (88)	1 (13)		

### Molecular detection

**Fig. 1:**
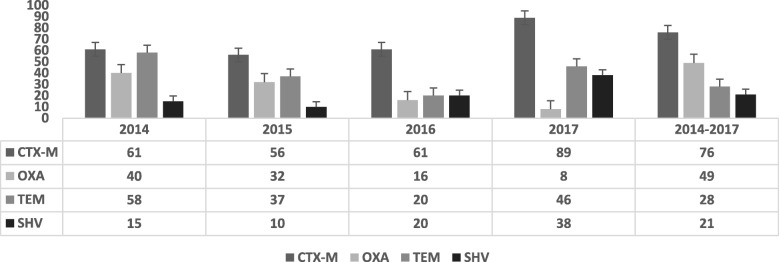
Year-wide prevalence of ESBL-encoding genes among clinical isolates. Percentages of ESBL-genes detected every year is tabulated

After screening, ESBL-producing isolates (*n* = 392) as detected by combination disc test were processed for the detection of *bla*_*CTX − M*_*, bla*_*SHV*_, *bla*_*TEM*_ and *bla*_*OXA*_ encoding genes by PCR. In Singleplex-PCR, *bla*_*CTX − M*_ genes were predominant 76% (*n* = 303) followed by *bla*_*OXA*_ 52% (*n* = 203), *bla*_*TEM*_ 28% (*n* = 109) and *bla*_*SHV*_ 21% (*n* = 82). Multiplex-PCR showed that *bla*_*CTX − M//SHV/TEM/OXA*_ and *bla*_*OXA/TEM/SHV*_ gene combination was present in 9% (*n* = 36) and 11% (*n* = 43) respectively. *bla*_*TEM/SHV*_ and *bla*_*TEM/OXA*_ combination was present in 13% (*n* = 51) and 27% (*n* = 105) respectively (Table 7, Fig. 1).

**Table 7:** Association of ESBL-production with type of specimen

Sample (*N* = 497)	Number (%)	ESBL-production		Chi-score	*p*-value
		Positive (%)	Negative (%)		
Urine	271 (55)	221 (82)	50 (18)	19.50840541	<0.0001
Pus	97 (20)	73 (75)	24 (25)		
Wound Swab	39 (8)	33 (85)	6 (15)		
Fluids and secretions	24 (5)	16 (67)	8 (33)		
Catheters and tips	17 (3)	11 (65)	6 (35)		
Blood	18 (4)	10 (56)	8 (44)		
Sputum	16 (3)	16 (100)	0 (0)		
High Vaginal Swab	7 (2)	6 (88)	1 (12)		
Others	6 (1)	6 (100)	0 (0)

**Table 8:** Gene variants obtained by amplicon sequencing in different isolates

Gene variant	Total (*n* = 392)	*Escherichia coli* (*n* = 321)	*Klebsiella pneumoniae* (*n* = 124)	*Enterobacter cloacae* (*n* = 20)	*Pseudomonas aeruginosa* (*n* = 24)	*Acinetobacter baumannii* (*n* = 8)	Chi-score	*p-value
*bla*_CTXM-1_	303 (76)	238 (74)	53 (43)	5 (25)	7 (29)	0 (0)	13.2333292	< 0.0001
*bla*_CTX-M-15_	260 (86)	204 (98)	48 (91)	3 (50)	5 (71)	0 (0)	6.336967046	0.0118
*bla*_OXA_	203 (52)	126 (39)	35 (28)	15 (75)	19 (79)	8 (100)	11.31364661	<0.0001
*bla*_OXA-1_	99 (49)	69 (55)	0 (0)	7 (50)	0 (0)	0 (0)	4.800612279	0.0284
*bla*_OXA-50_	7 (3)	0 (0)	0 (0)	0 (0)	7 (100)	0 (0)	3.997740394	0.0456
*bla*_OXA-144_	2 (1)	0 (0)	0 (0)	0 (0)	0 (0)	2 (25)	32.84026642	<0.0001
*bla*_OXA-23_	4 (2)	0 (0)	0 (0)	0 (0)	0 (0)	4 (50)	65.68053525	<0.0001
*bla*_OXA-371_	2 (1)	0 (0)	0 (0)	0 (0)	0 (0)	2 (25)	32.84026642	<0.0001
*bla*_OXA-58_	2 (1)	0 (0)	0 (0)	0 (0)	0 (0)	2 (25)	32.84026642	<0.0001
*bla*_OXA-68_	2 (1)	0 (0)	0 (0)	0 (0)	0 (0)	2 (25)	32.84026642	<0.0001
*bla*_OXA-94_	2 (1)	0 (0)	0 (0)	0 (0)	0 (0)	2 (25)	32.84026642	<0.0001
*bla*_TEM_	109 (28)	82 (29)	23 (19)	4 (20)	0 (0)	0 (0)	16.56736583	<0.0001
*bla*_TEM-1B_	69 (34)	76 (33)	19 (83)	1 (25)	0 (0)	0 (0)	1.942065018	
*bla*_SHV_	82 (21)	10 (3)	65 (52)	7 (35)	0 (0)	0 (0)	13.56652163	<0.0001
*bla*_SHV-10_	5 (6)	1 (10)	4 (8)	0 (0)	0 (0)	0 (0)	5.566456898	0.0183
*bla*_SHV-11_	47 (57)	0 (0)	47 (89)	0 (0)	0 (0)	0 (0)	22.26584202	<0.0001
*bla*_SHV-1_	13 (16)	0 (0)	13 (25)	0 (0)	0 (0)	0 (0)	16.69937791	<0.0001
*bla*_SHV-27_	4 (5)	0 (0)	4 (8)	0 (0)	0 (0)	0 (0)	5.566456898	0.0183
*bla*_SHV-28_	4 (5)	0 (0)	4 (8)	0 (0)	0 (0)	0 (0)	5.566456898	0.0183
*bla*_SHV-83_	24 (29)	0 (0)	24 (45)	0 (0)	0 (0)	0 (0)	5.566456898	0.0183
Gene combinations								
*bla*_CTX-M-15_ + *bla*_OXA-1_	71 (14)	55 (60)	5 (42)	2 (10)	0 (0)	0 (0)	5.44065331	0.0197
*bla*_OXA-1_ + *bla*_TEM-1B_	30 (6)	22 (20)	6 (50)	1 (5)	0 (0)	1 (13)	0.485769791	
*bla*_CTX-M-15_ + *bla*_TEM-1B_	44 (9)	33 (7)	10 (8)	0 (0)	0 (0)	1 (13)	1.009389671	
*bla*_CTX-M-15_ *+ bla*_SHV-11_	4 (3)	0 (0)	4 (33)	0 (0)	0 (0)	0 (0)	19.46666667	<0.0001
*bla*_CTXM-15_ + *bla*_OXA-1_ + *bla*_TEM-1B_	28 (4)	22 (7)	5 (4)	0 (0)	0 (0)	1 (25)	0.234071093	

*only *p*-values <0.05 are shown

Amplicon sequencing and subsequent analysis indicated *bla*_*CTX − M −* 15_ 86% (*n* = 260) was prevalent among *bla*_*CTX − M −* 1_ group. *bla*_*OXA −* 1_ 49% (*n* = 99) were found among *bla*_*OXA*_ amplicons and *bla*_*TEM −* 1*B*_ 63% (*n* = 69). 83% (*n* = 190) of *E. coli had bla*_*CTX − M −* 15_ followed by *bla*_*OXA −* 1_ 55% (*n* = 69) and *bla*_*TEM −* 1*B*_ 33% (*n* = 76). *Klebsiella spp.* contained *bla*_*CTX − M −* 15_ 67% (*n* = 36) followed by *bla*_*SHV −* 11_ 89% (*n* = 47) and bla_TEM *−* 1*B*_ 34% (*n* = 19). While, *Pseudomonas spp.* and *Acinetobacter baumannii* had variants of OXA (*bla*_*OXA −* 50_, *bla*_*OXA −* 144_, *bla*_*OXA −* 23_, *bla*_*OXA −* 371_, *bla*_*OXA −* 58_, *bla*_*OXA −* 68_ and *bla*_*OXA −* 94_) (Table 8).

## Discussion

Extensive use of antibiotics has resulted in resistance against variety of antibiotics including cephalosporins. They affect countries all over the world but control and prevention of ESBL-producers is severely compromised in underdeveloped countries [31–33].

Here, high prevalence of third generation cephalosporin resistant isolates (50%) was observed which has subsequently increased by 1.13-fold from 2014 to 2017. This high resistance also indicates high selection pressure for third generation cephalosporin resistant isolates [34]. This increase of resistance is worrisome as we are left with few treatment options including cephalosporins. Widespread usage of antibiotics might be the factor of such increase in resistance in our hospital settings [16, 35].

*E. coli* had high 3GC-R burden compared to *Klebsiella* spp. and *Enterobacter* spp. Bari et al., reported similar findings in a study conducted in 2013 in Lady Reading Hospital Peshawar [36]. These results are comparable to findings in Tanzania where 45% ESBL-producers have been reported [37]. Similar findings from different regions of the world were observed as previously studied [38, 39]. Nahid et al., reported very high prevalence of ESBL-producers (87.5%) but this is because she worked on Metallo-*β*-lactamase producers which are highly resistant organisms [40].

ESBL infectivity rate in males was moderately high as compared to females. This rate is quite similar to the rate reported by Afirdi et al. [41]. In our study ESBL infections were significantly higher in the mean age group of 41-60 years whereas, high infection rates have been reported in old age individuals who are immuno-compromised and hence, more prone to infections [42]. We have found isolates originating from females were more frequent ESBL-producers. According to many reports males have significantly higher rates of hospital-acquired infection and community-acquired infections are more prevalent in females [42–46]. These findings represent that males are more often exposed to the hospital settings compared to the females.

Studies indicated prevalence of ESBL-producers is variable in different regions of world as detected by phenotypic detection tests [47–50]. DDST determined only 54 % strains as ESBL-producers while CDST determined 79 % as ESBL-producers. Ejaz et al., reported similar detection efficiency of CDST as we reported here [17]. Prevalence of ESBL-producing isolates is quite higher than from other parts of the world including India (42.3 %), Bangladesh (37.8 %). Dalela et al., reported 90 % sensitivity of CDST for the detection of ESBL-producers [51]. E-test revealed that 61 % strains were ESBL-producers while 39 % remained non-determined by this technique. Mohanty et al. also reported 61 % positivity rate for ESBL-producers by E-test technique [52]. Such discrepancies between susceptibility data and phenotypic test results have increased the demand for more sensitive methods of ESBL-producer detection for implementation into routine susceptibility testing procedures.Despite of high resistance burden of ESBL-producers, the usage of molecular detection methods is not very common. A recent meta-analysis describes only 11% studies that reported PCR-based detection methods for screening of ESBL-producers in Pakistan [20]. Lack of knowledge and technical staff triggers the use of PCR-based methods as it is the rapid and reliable method of ESBL-producer detection [8]. It seems that *bla*_*CTX − M*_ is predominant genotype in this region of the world. Another study from Pakistan indicated 72% of isolates had *bla*_*CTXM −* 15_ gene which was lower than prevalence of *bla*_*CTX-M*_ gene found in this study [16]. Few studies from other parts of world have shown different prevalence of *bla*_*CTX-M*_ gene among isolates including 84.7% (Chile), 98.8% (China) and 13.6% (Tanzania) [53–55]. We observed *bla*_*TEM*_ and *bla*_*OXA*_ genes were less common in our settings with 50% prevalence. Report from Hamad Medical Corporation, Qatar stated that CTX-M group has evolved through mutations in *bla*_*TEM*_ and *bla*_*SHV*_ genes and is recent endemic [56].

-*Acinetobacter baumannii* isolates had OXA variants (*bla*_*OXA −* 23*,*58_ and others) which are carbapenemase-encoding genes [57]. These variants have previously been isolated from France, Spain and Turkey which indicates the global spread [50]. *bla*_*OXA −* 23_ was amplified from pan-drug resistance *A. baumannii* only which is in accordance with our results [58]. But these *Acinetobacter baumannii* isolates did not carry any of the ESBL-encoding genes which terminate the co-existence of carbapenemase and ESBL-encoding genes. This is in accordance with already published article which states no significant relation between both groups [59]. Appearance of different variants might provide extra advantage for these isolates to spread them and complicate the therapeutics.

With the passage of time increase in co-resistance of different ESBL-producing genes is worrisome as co-existence of multiple genes hinders the detection of ESBL-producers and complicates the treatment strategy for clinicians. Moreover, high plasmid burden was found these plasmids are involved in gene-transfer and they also carry additional antibiotic resistance genes along with *β*-lactam antibiotics.

## Conclusions and Recommendations

In conclusion, *bla*_CTX−M_-type ESBL-producing genes and *bla*_OXA_-type narrow spectrum-β-lactamases are prevalent among the isolates in our health care settings. Isolates had high resistance towards cephalosporins. Resistance towards cephalosporins and carbapenems has increased many folds during study period. Co-expression of multiple genes complicates the treatment strategy. *bla*_*CTXM−*15_, a pandemic genotype is quite prevalent and their plasmid association is a big thread for the community. There is a dire need for efficient molecular diagnostic tools for the detection of *bla* genes at laboratory level.

## Abbreviations

CDST: Combination disc test
DDST: Double disc synergy test
ESBL: Extended-spectrum *β*-lactamases
ESBLs: Extended-spectrum-β-lactamase-producing strains
E-Test: Epsilometric test
MAR: Multiple- antibiotics resistance
MIC: Minimum inhibitory concentration
